# Fully Transparent Gas Sensor Based on Carbon Nanotubes

**DOI:** 10.3390/s19204591

**Published:** 2019-10-22

**Authors:** Florin C. Loghin, Aniello Falco, Jose F. Salmeron, Paolo Lugli, Alaa Abdellah, Almudena Rivadeneyra

**Affiliations:** 1 Institute for Nanoelectronics, Technical University of Munich, 80333 Munich, Germany; florin.loghin@tum.de (F.C.L.); alaa.abdellah@tum.de (A.A.); 2 Faculty of Science and Technology, Free University of Bolzano, 39100 Bolzano-Bozen, Italy; aniello.falco@unibz.it (A.F.); paolo.lugli@unibz.it (P.L.); 3 Pervasive Electronics Advanced Research Laboratory (PEARL), Department of Electronics and Computer Technology, University of Granada, 18071 Granada, Spain; jfsalmeron@ugr.es

**Keywords:** ammonia, CO_2_, single-walled, spray deposition, transparent electrodes

## Abstract

In this paper, we demonstrate the feasibility of realization of transparent gas sensors based on carbon nanotubes (CNTs). Both sensing layer and electrodes consist of CNTs deposited by spray deposition. The transparent sensor—with a transmittance higher than 60% in both sensing layer and electrodes—is characterized towards NH_3_ and CO_2_ and compared with a reference sensor with the same active layer but evaporated Au electrodes. In particular, the sensitivity towards NH_3_ is virtually identical for both reference and transparent sensors, whereas the transparent device exhibits higher sensitivity to CO_2_ than the reference electrode. The effect of the spacing among consecutive electrodes is also studied, demonstrating that a wider spacing in fully CNT based sensors results in a higher sensitivity because of the higher sensing resistance, whereas this effect was not observed in gold electrodes, as their resistance can be neglected with respect to the resistance of the CNT sensing layer. Overall, the transparent sensors show performance comparable—if not superior—to the traditionally realized ones, opening the way for seamlessly integrated sensors, which do not compromise on quality.

## 1. Introduction

One of the new trends in technology is transparent electronics (TE) whose main feature is the development of invisible circuits. This technology based on transparent materials will allow us to define circuitry on any surface, making easier the development of the paradigm of the Internet of Everything (IoE) without interfering in the appearance and usage of the objects/entities where the devices are located. The realization of electronics, which is seamlessly integrated in daily objects, as well as in professional tools, might lead to a new set of applications, which would have been unthinkable just a few years ago. Augmented reality headsets, cellular antennae integrated into building’s windows [1] and hybrid wearable electronics [2] are just a few examples of commercial products enabled by the improvements in the field of transparent electronics. The first factor defining TE devices is, obviously, their capability of being transparent, which is normally quantified in terms of their transmittance spectra. This quantity is defined as the fraction of incident light at a specific wavelength that passes through the sample. The first advances of this technology were in the field of transparent conductive materials leading to liquid crystal displays (LCDs) [3] and solar cells [4] based on them. From a historical point of view, we are currently in the second generation of TE, where some devices—mainly fabricated with traditional techniques—such as thin film transistors (TFT) [5,6], ultraviolet (UV) sensors [7] and imagers [8] have been developed but the research is constantly evolving towards new applications [9,10].

The main achievements in TE are analyzed below, starting with thin-film electronic layers, followed by single devices and ending with electronic systems. Many of the advances and efforts are focused on the development of transparent electrodes for optoelectronic devices in order to optimize their performance. In this scenario, indium tin oxide (ITO) is the most used material for its stability and conductivity. However, its high cost has led researchers to further investigate other conductive materials. In particular, flexible and transparent electronics attract a lot of attention, where metal nanowires and conductive materials have opened a wide range of applications. Lipomi et al. reported the electronic and morphological characteristics of poly(3,4-ethylenedioxythiophene) polystyrene sulfonate (PEDOT:PSS) deposited on stretchable poly(dimethylsiloxane) (PDMS) substrates [11]. They explored how the formulation of conductive PEDOT:PSS together with surface treatment of PDMS influence the resistance of the conductive film and its evolution when it is exposed to various stretch and strain conditions. They obtained wave-like buckles formed after the first stretch (>10%), making the film reversibly stretchable. This result only confirms that conductive PEDOT:PSS films can be moderately stretched while maintaining their transparency. But they can only support 30% uniaxial strain. Also, their resistance is relatively high to define efficient interconnects and the manufacturing technique, spin coating, is not scalable and only allows full coating of the surface. Bobinger et al. presented fabrication of homogeneous and large-area silver nanowire (AgNW) by spray deposition [12]. They showed how the number of layers and hence the deposition time modulates the thermal and electrical properties of these thin films and the application of these films as transparent heaters. Another interesting material is carbon nanotubes (CNTs). These films show lots of desirable characteristics for printable TE, such as high conductivity, high carrier mobility, optical transparency and flexibility [13]. The main problem of this material is its environmental stability over time, that is to say, it is difficult to control and to predict its variations over time due to the fact that CNTs present high sensitivity to many environmental conditions, such as temperature, relative humidity or gas concentration [14,15,16]. On another front, there have been many efforts to obtain transparent films based of graphene whose outstanding electrical, mechanical and chemical properties make it a perfect candidate for flexible electronics. Bae et al. demonstrated an efficient method based on roll-to-roll production and chemical vapor deposition (CVD) for the synthesis, transfer and doping of thin film graphene [17]. Although reproducible films were obtained, it is not possible to do patterning of the films and there are manufacturing processes incompatible with printed electronics. A different and recent solution was presented by Kang et al. [18], based on ‘polymer-metal hybrid electrodes’ where a customized conductive and flexible polymer is prepared. This polymer exhibits both high transparency (above 90%) and low sheet resistance (tens of Ω/sq.). Although this optoelectronic performance is desirable for printable TE, the manufacturing technique (CVD) only allows the full coating of layers, limiting the application of this material in real applications.

Regarding devices, the main scientific contributions are in the direction of transparent TFT and physical sensors. Lee et al. presented a transparent bending-insensitive pressure sensor [19]. They reported the fabrication of extraordinarily small bending-sensitive, ultra-flexible, optically transparent and resistive-type pressure sensors using composite nanofibers. This sensor provides an accurate measurement of only the normal pressure without suffering from any mechanical deformation. These excellent mechanical and transparent properties lack a fabrication process compatible with printed electronics and they can only fabricate films. Actually, the electrodes used to test this sensor are made from sputtered ITO and the sensing layer was deposited by electrospinning, requiring clean room processes. In the direction of pressure sensors, a transparent and low-power sensor matrix based on coplanar-gate graphene transistors was reported by Sun et al. [20]. They made use of different fabrication techniques not combinable at large-scale and with incompatibilities in terms of temperature and atmosphere conditions. Also, Trung et al. [21] described a transparent and stretchable temperature sensor for body-attachable wearable electronics, showing also its response to pressure. In their work, non-controllable and non-patterning procedures (spin coating and mask etching) were used again to define the devices, limiting their use in the definition of circuitry layouts. Another example of this kind of device is the fabrication of transparent thin-film transistors with elastomeric behavior described by Liang et al. [22]. The entire fabrication was done with solution-based techniques, although they employed drop casting as fabrication method, with difficult control of the quality of the deposition and modest up-scalability. There are some examples of printed CNT films in the literature [23,24] using different fabrication processes.

In this article, we show a transparent gas sensor entirely fabricated by spray deposition, with performance comparable to similar works obtained with opaque layers and less scalable deposition techniques. Furthermore, as both the electrodes and the sensing layer are made of CNTs, we reduce the need of precious materials such as gold or indium, which are commonly employed in similar applications. To attain the goal, we first characterize the conductance of non-patterned bare CNTs, in order to find a compromise between the achieved resistance and the sheet resistance for the electrode layer. Subsequently, we compare the performances of sensors with differently spaced fingers, in order to find the optimal geometrical structure. Ultimately, we show how the most effective combination presents performances comparable—and even superior, in certain conditions—to sensors fabricated with gold electrodes. Hence, we demonstrate how our approach allows for facile and highly scalable depositions, while enabling the obtainment of semitransparent sensors whose performances are not hindered by the technical solutions employed for fabrication.

## 2. Materials and Methods

### 2.1. Materials and Fabrication Process

For the sensor fabrication, a two-step approach was employed. The initial step was the deposition and patterning of the electrodes followed by a deposition of the active channel on foil (Kapton HN). For the reference sensor, gold contacts were evaporated by physical vapor deposition onto a pre-patterned photoresist layer. Following a lift-off process, the resulting interdigitated electrode structure was finalized. Sensors with 50 µm, 100 µm and 200 µm spacing were developed. For the CNT-based electrodes, CNTs (single walled, HANWHA Nanotechnologies), from an in house made aqueous dispersion stabilized with a dispersant, sodium carboxymethyl cellulose (CMC) [25], were deposited with various layer numbers using an industrial atomizing spray valve mounted into an automated motion platform. For further reference on the spraying parameters and strategy, please consult [26]. Post deposition the samples were placed in dilute HNO_3_ overnight to remove the dispersant. The samples were subsequently annealed at 100 °C to desorb any excess HNO_3_ and washed with deionized water. In order to characterize the resulting layer for the opto-electrical properties, unpatterned samples, on foil, were fabricated with various layer numbers. For the patterning of the electrodes, the film was masked with a photoresist and with the aid of oxygen plasma; the CNTs were selectively etched to result in the same interdigitated structure as with the gold electrodes. The masking photoresist was removed using an acetone bath. The film thickness of each layer is 48 nm, 60 nm, 74 nm and 97 nm, for 20, 25, 30 and 40 layers respectively.

**Figure 1:**
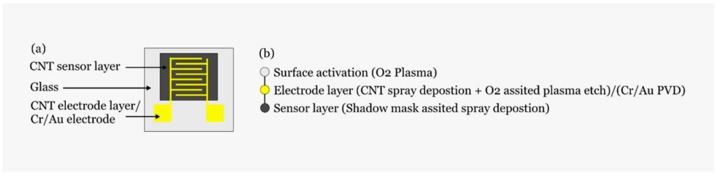
(**a**) Sensor schematic; (**b**) fabrication flow.

The processing of the active sensing layer was identical for both sensors. The CNTs (single walled, Carbon Solution Inc., Riverside, CA, USA) were deposited, with the aforementioned spray valve/stage system, from an aqueous solution using CMC as a dispersant. Due to the low CNT density required, the solution was diluted down at a ratio of 1:14 solution:deionized water. Similarly, to the electrode structures the samples were placed in HNO_3_ overnight, annealed at 100 °C and washed with deionized water. Figure 1 depicts a schematic of the resulting sensor as well as the process flow for its realization.

### 2.2. Characterization

The sensor module is composed by the gas sensor, placed on a carrier glass together with a temperature sensor (Pt100) for in-situ temperature monitoring and a Peltier heating element for temperature control.

Sensors were characterized by measuring the relative change in resistance with respect to the NH3 and CO2 concentration. In this sense, the voltage drop across its terminal was measured under a constant sensing current. Before exposing the sensor towards the gases, the initial resistance of the device was monitored for 1000 s in order to determine a baseline for this sensor. Once a stable initial resistance was achieved, we introduced the sensor module inside the gas chamber and evaluated its response by exposing it to different gas concentrations for each of the analyzed gases. The sensor performance was evaluated through its normalized resistance (*NR*), defined in (1) as the relative change in resistance, where *R_i_* and *R_f_* are the initial and final values of resistance in an exposure cycle, respectively.

(1) $$NR=\frac{R_{f}-R_{i}}{R_{i}}$$

The concentration range for NH_3_ was between 10 ppm and 100 ppm, achieved by diluting the test gas with nitrogen as carrier gas at ambient conditions, whereas for CO_2_ the tested concentrations were 1000 to 5000 ppm with compressed air as carrier gas at ambient conditions. We selected these two gases because of their industrial and environmental interest [27,28] and because response of CNT-based devices have been widely studied towards them [29,30,31]. A measurement cycle consisted of an exposure interval followed by a recovery interval. The exposure to the test gas along with the carrier gas was set to a constant flux of 200 mL/min for 300 s at ambient temperature. Recovery was done by heating the sensor module to 80 °C. After that, the high flux (1000 mL/min) was kept for another 300 s at ambient temperature to facilitate the cooling of the sensor and remove any residual test gas molecules from the chamber. The last step in the recovery stage was a constant flux of the carrier gas for 300 s at ambient conditions in order to reestablish the initial value of resistance before a new exposure cycle.

The sheet resistance measurements were performed with a four-point probe head from Jandel connected to a source measuring unit (Keysight B2901A). All measurements were taken applying a constant current of 1 mA. The transmittance of the CNT films were measured as described in [32]. In order to compare this transparent gas sensor to a conventional one, we used the same geometry and sensor architecture but with evaporated gold electrodes as reference sensor with a spacing between consecutive fingers equal to 200 µm, similar to the one presented in [33].

## 3. Results and Discussion

### 3.1. Optical and Electrical Characterization

**Figure 2:**
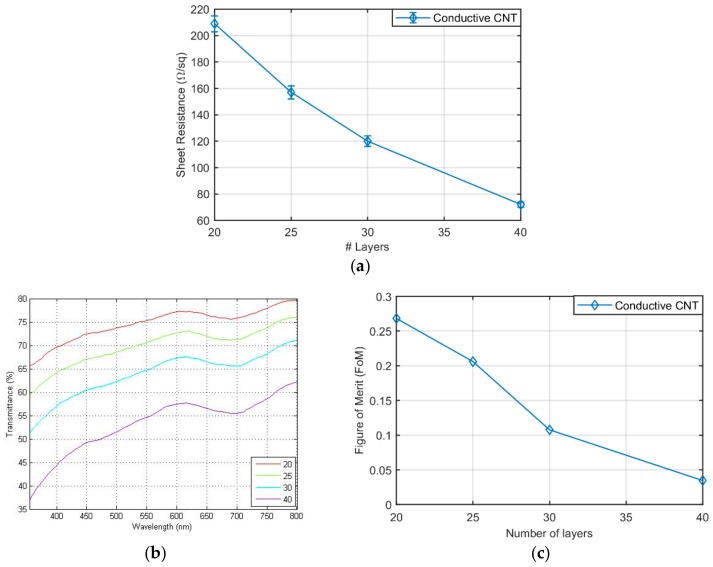
(**a**) Sheet resistance of carbon nanotube (CNT) films with regard to the number of CNT layers sprayed. Corresponding to 48 nm, 60 nm, 74 nm and 97 nm film thickness, for 20, 25, 30 and 40 layers, respectively; (**b**) optical transmittance of CNT layers for electrodes definition; (**c**) electro-optical figure of merit as a function of the number of layers.

The experimental values of sheet resistance and transmittance in the range of 350 nm to 800 nm for films of different thickness are shown in Figure 2b, respectively. Visibly, the higher number of sprayed layers is, the lower the sheet resistance and the transmittance are. Transmittance values above 60% enable the use of such networks as semi-transparent electrodes for optoelectronic applications [34]. Therefore, it is necessary to find a trade-off between sheet resistance (desired to be below 150 Ω/sq to reduce the impact of the series resistance) and transmittance (expected to be as high as possible for transparent electrodes).

The usual approach for the solution of this trade-off is to look at the electro-optical figure of merit (*FoM*) [35], defined as a function of the transmittance at 550 nm and the sheet resistance *R_s_* as:

(2) $$FoM=1000\times \frac{{\left(T_{550}\right)}^{10}}{R_{s}}$$

Figure 2c presents the results, and shows how the highest *FoM* is obtained for the thinner layers. An acceptable compromise (i.e., highest *FoM* with resistance below 150 Ω/sq) is found for the spray deposition of 30 CNT layers, exhibiting 120 Ω/sq at 65% at 550 nm, similar to what has been achieved in previous works, [24,32] and it was chosen as preferred electrode thickness. Notice that the sheet resistance of evaporated Au electrodes is circa 0.2 Ω/sq with virtually no transmittance in the whole analyzed spectrum.

In the case of the CNT sensing layer, we fixed the number of layers to four because we already proved that this number provides a good compromise between sensitivity and reproducibility [25].

### 3.2. Gas Characterization

**Figure 3:**
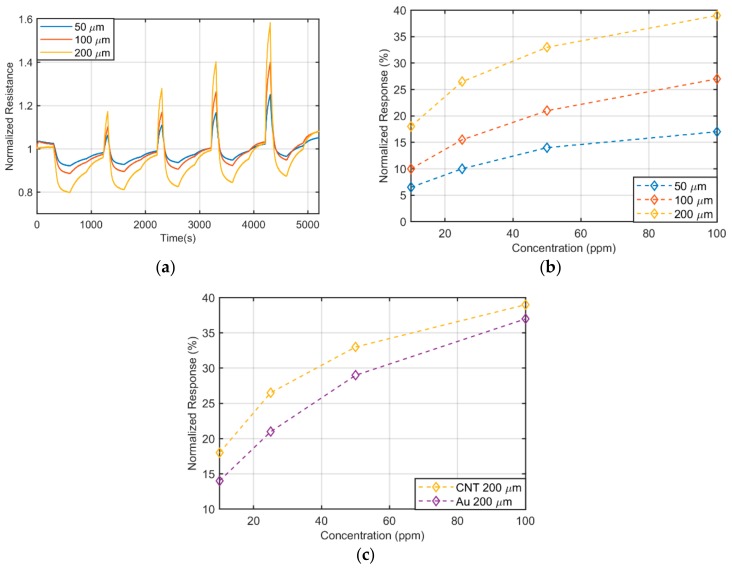
(**a**) Time response of fully transparent sensor to NH_3_; (**b**) calibration curves of fully transparent sensor for NH_3_; (**c**) calibration curves towards ammonia for 200 µm spacing for Au and CNT electrodes.

Figure 3a depicts the temporal response of the resistance under exposure of 10 ppm, 25 ppm, 50 ppm and 100 ppm, of NH_3_, during 300 s of exposure for CNT-electrodes with different distance among consecutive fingers (spacing). Figure 3b illustrates the normalized response with respect to NH_3_ concentration for the different geometries tested. From this graph, we extracted the sensitivity of this sensor, defined as the slope of the linear regression curve. Figure 3c compares the response of the fully-CNT gas sensor with the highest response of an identical Au based sensor. It can be observed that both curves are similar, proving that the Au-evaporated electrodes can be substituted by the CNT-based ones without affecting the sensor behavior. In addition to this, the substitution of the Au electrodes does not seem to affect the recovery time and time constant of the system (data not shown).

The sensitivity found in the range of 10–100 ppm for the different sensors is presented Table 1. Remarkably, the wider the electrode spacing, the higher the sensitivity, and for the considered geometry and material set, a finger spacing of 200 µm is the best candidate for gas sensing. The reason behind this result resides in the increased resistance of the sensing layer with respect to the electrodes. A static approximation of the resistance change of the sensor as a response to a gas concentration (*NR*(*c*)) can be written in terms of the electrode resistance *R_e_* and the sensing resistance *R_s_* as:

(3) $$R=R_{e}+R_{s}\left(1+NR\left(c\right)\right)$$

**Figure 4:**
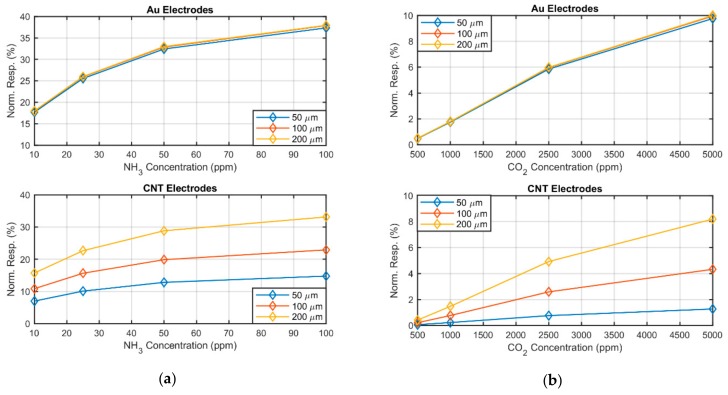
(**a**) Calculated normalized resistance change to NH_3_ and (**b**) calculated normalized resistance change to CO_2_ for sensors fabricated with Au electrodes (**top**) and CNT electrodes (**bottom**). The discrepancy in response is directly related to the non-negligible electrode resistances, which are 20 Ω for the Au and circa 5.5 kΩ for CNT.

Assuming the electrode resistance to be the same for all the fabricated sensors and its change to be negligible with respect to the change of the sensing resistance, it is evident how higher sensing resistance (i.e., wider spacing) leads to higher sensitivity. This effect was not observed in gold, as the resistance of the electrodes can be neglected with respect to the resistance of the sensing layer. Figure 4 presents the theoretical resistance changes due to the presence of different gas concentrations, providing a graphical intuitive justification to the observed phenomenon.

In particular, the electrodes’ resistance was circa 20 Ω for the Au and circa 5.5 kΩ for CNT. Considering these electrode resistances, we extrapolated an average film resistance for the different structure width, equal to 0.8 kΩ, 4.4 kΩ and 25 kΩ, for 50 μm, 100 μm and 200 μm, respectively. This simple model shows how, on the one hand, the spacing is particularly important for high resistance films, and on the other hand that the effect would eventually saturate for particularly wide electrode spacing. Ideally, the normalized response for CNT electrodes would converge to one of the Au electrodes, if the ratio of the resistance of the sensing layer to the electrodes was sufficiently large.

The choice of an even higher electrode distance, however, would affect the reproducibility of the overall resistance of the sensor [36], while, at the same time, rendering the measurement with low-cost equipment more noisy and unstable.

**Figure 5:**
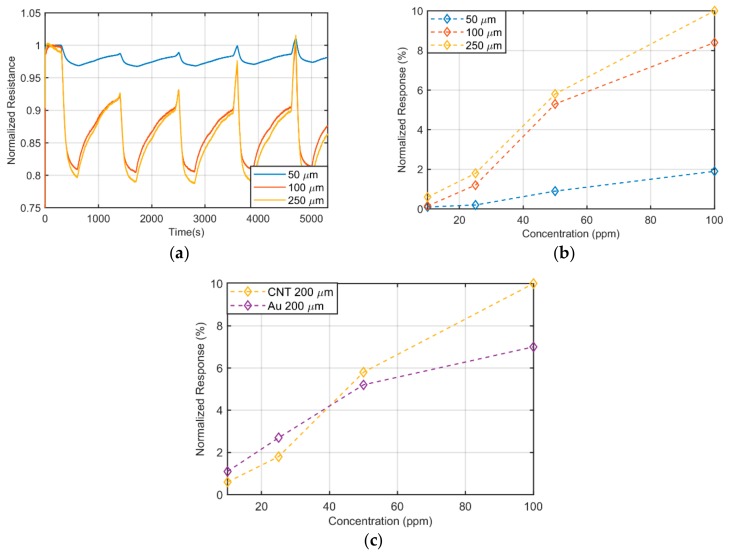
(**a**) Time response of fully transparent sensor to CO_2_; (**b**) calibration curves of fully transparent sensor for CO_2_; (**c**) calibration curves towards carbon dioxide for 200 µm spacing for Au and CNT electrodes.

Given the importance of monitoring CO_2_ both in industrial plants and living environments, the realization of semi-transparent CO_2_ sensors would have capital importance. For this reason, we repeated a similar characterization approach for CO_2_, showing the temporal response of the sensor (Figure 5a), its response to the tested concentrations (Figure 5b) and its comparison with a sensor with Au electrodes (Figure 5c). The sensitivity, linearity and initial value of resistance are detailed in Table 2. Once more, the sensitivity of the sensor at higher concentration is slightly better in the case of fully-CNT gas sensor. This effect can be related to the fact that in the carbon-based sensor also the electrodes are sensitive to the presence of gas molecules, although their response to the gas is different, because of the different network density. Interestingly, for CO_2_ exposure, the effect of different spacing is slightly different in magnitude from what has been previously observed for NH_3_: The difference between the calibration curves for 100 μm and 50 μm is much larger than the difference between 200 μm and 100 μm. This observation, coupled with the fact that CNT electrodes perform better than Au electrodes for CO_2_ exposure, seems to indicate that the effect of this gas on the electrodes themselves is not negligible. At higher concentrations, CO_2_ is able to induct a big change in the electrodes’ resistance, which results in a distance-insensitive increased normalized resistance change.

As demonstrated by the results, these transparent devices not only can be used in certain applications where transparency is mandatory or desirable but also, they provide a higher response than those made with gold electrodes, especially in the case of CO_2_. Therefore, they are interesting even in those applications where a transparent sensor is not required, providing also a solution processable device that could be in principle fabricated on any surface. As a final remark, it must be noted how a classic problem of CNT-based gas sensors is still present in these devices: The lack of selectivity. This becomes an issue when the sensors are employed in environments where both species are potentially present, however, the solution to this issue lies beyond the scope of this work. For the interested reader, nevertheless, we have proposed in the past solutions which could be employed with a minor increase in process complexity. This drawback could, for instance, be overcome by the addition of metal nanoparticles (NPs) to the CNT ink as shown in [37]. Due to the low quantity of NPs required to tune the selectivity, the transparency of the described devices would not be altered.

## 4. Conclusions

In this paper, we show the feasibility of fabricating low-cost transparent gas sensors fully fabricated with CNT. In particular, the electrodes are first deposited using spray deposition. To achieve high conductivity, several layers of unsorted CNTs are sprayed. Subsequently, the active layer based on a similar CNT solution, but diluted is sprayed on top of the electrodes. The transmittance of the fabricated device is limited by the thicker layer required for the electrodes, therefore, a trade-off between sheet resistance and transmittance is mandatory. We selected 30 spray layers that result in transmittance above 60% and sheet resistance below 120 Ω/sq.

The transparent device presents a very similar initial resistance to a reference sensor made with the same sensing layer but with evaporated Au electrodes. With respect to NH_3_, both reference and fully CNT sensors exhibit similar sensitivities in the range of concentrations studied (0.22% VR/ppm for Au electrodes and 0.25% VR/ppm for CNT electrode device). In the case of CO_2_, the transparent sensor has almost double the sensitivity (0.0021% VR/ppm) than the reference device (0.0012% VR/ppm), since, in the former case, both the sensing layer and the electrodes change their resistance when exposed to gas. 

We also analyzed the effect of the electrode spacing in the sensor performance, showing that for full CNT sensors, the larger the distance between consecutive electrodes is, the higher the sensitivity. We attribute this effect to the non-negligible contribution of the CNT electrodes resistance to the overall resistance of the sensor. 

Given the proven effectiveness of CNT networks as sensing layers for a number of different physical and chemical quantities, we believe that the reproduction and improvement of the approach presented in this contribution could be extended to many sensor classes. For this reason, we believe that this work could be seminal for the obtainment of inexpensive transparent sensors and actuators, which will help shape the new generation of transparent electronics.

## Figures and Tables

**Table 1:** Sensitivity towards NH_3_.

Spacing (µm)	Electrode Material	Initial Resistance (kΩ)	Sensitivity (%norm.Response/ppm NH_3_)	Linearity (R^2^)
50	CNT	6.16	0.1164	0.9621
100	CNT	10.29	0.1831	0.9627
200	Au	27.80	0.2165	0.9687
	CNT	27.09	0.2484	0.9428

**Table 2:** Sensitivity towards CO_2_.

Spacing (µm)	Electrode Material	Initial Resistance (kΩ)	Sensitivity (%norm.Response/ppm CO_2_)	Linearity (R^2^)
50	CNT	4.00	0.000386	0.9973
100	CNT	12.95	0.001856	0.9870
200	Au	34.74	0.001230	0.9596
	CNT	31.55	0.002114	0.9930
